# Supplementary material for: Synthesis of Terminal-Alkylated PEGs with Imine Spacer to Form Iminium Mono-Ion Complexes for pDNA Delivery into Skeletal Muscle
Source: Pharmaceutics. 2025 Aug 13;17(8):1054. doi: 10.3390/pharmaceutics17081054 (PMC12389348; doi:10.3390/pharmaceutics17081054)
Supplement: Supplementary file 1 [file pharmaceutics-17-01054-s001.zip › pharmaceutics-3789740-supplementary.pdf]

Supplementary Materials

Synthesis of Terminal-Alkylated PEGs with Imine Spacer to Form Iminium Mono-Ion Complexes for pDNA Delivery into Skeletal Muscle

Riku Oba[1], Yoko Endo-Takahashi[2], Yoichi Negishi[2], and Shoichiro Asayama[1,*]

1 Department of Applied Chemistry, Tokyo Metropolitan University, Hachioji, Tokyo 192-0397, Japan
2 School of Pharmacy, Tokyo University of Pharmacy and Life Sciences, Hachioji, Tokyo 192-0392, Japan

* Email: asayama-shoichiro@tmu.ac.jp

*

(A)

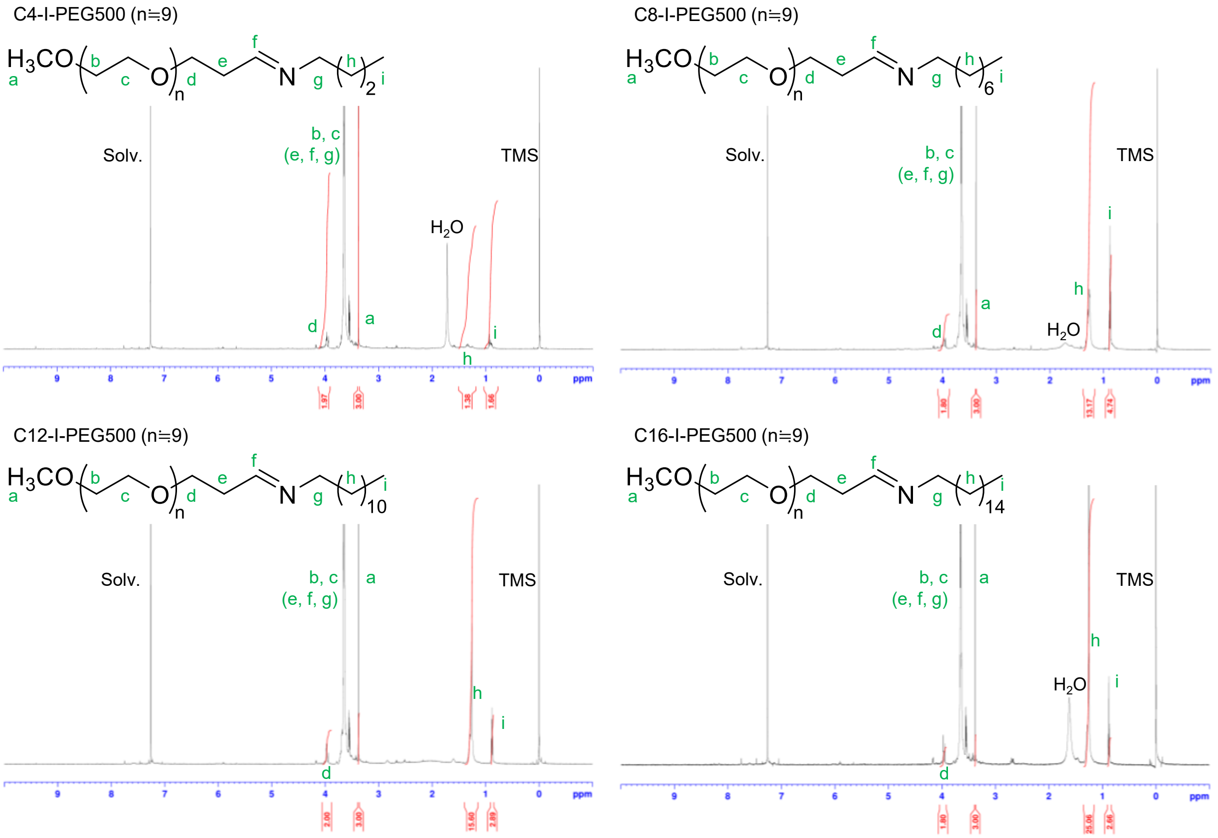
C4-I-PEG500 (n≒9)
C8-I-PEG500 (n≒9)
C12-I-PEG500 (n≒9)
C16-I-PEG500 (n≒9)
H3CO
N
Solv.
TMS
H2O
b, c
(e, f, g)
ppm

(B)

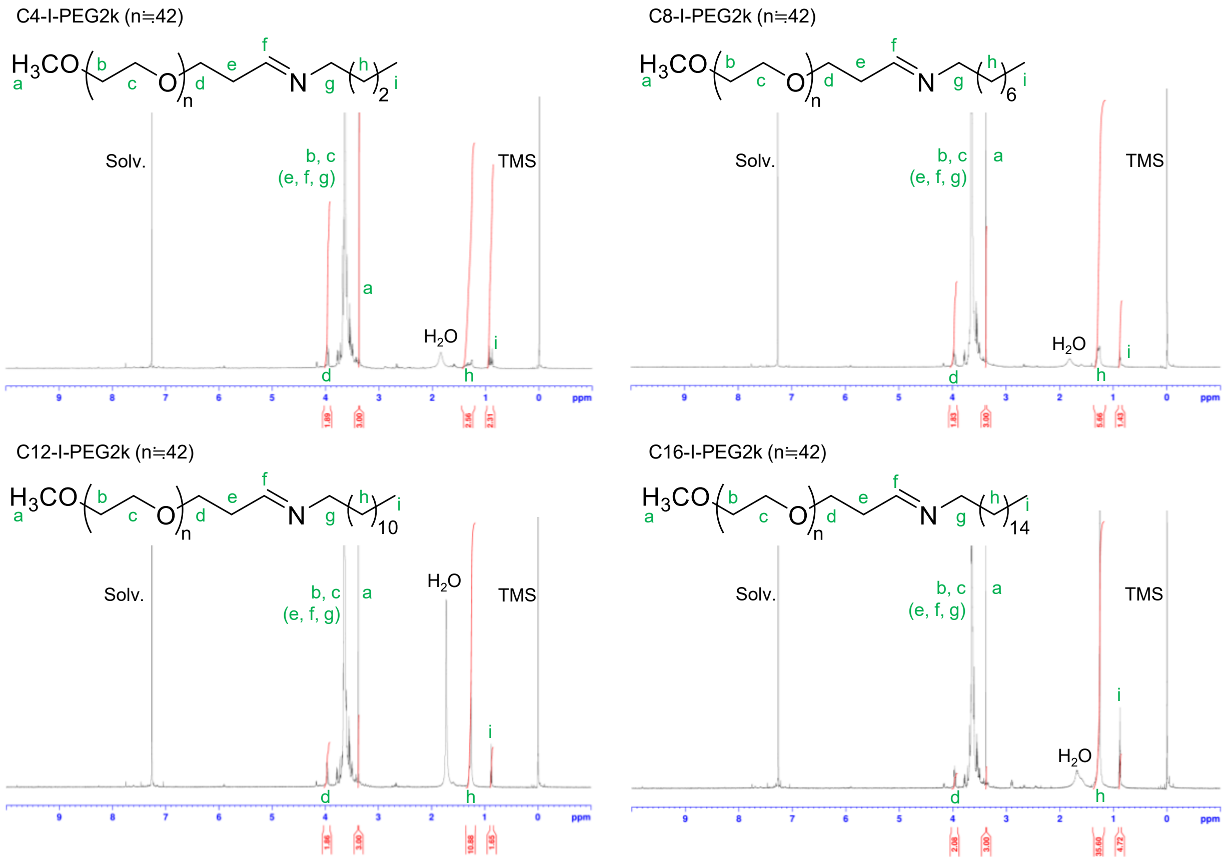
C4-I-PEG2k (n≒42)
C8-I-PEG2k (n≒42)
C12-I-PEG2k (n≒42)
C16-I-PEG2k (n≒42)
H3CO
N
Solv.
TMS
H2O
b, c
(e, f, g)
ppm

(C)

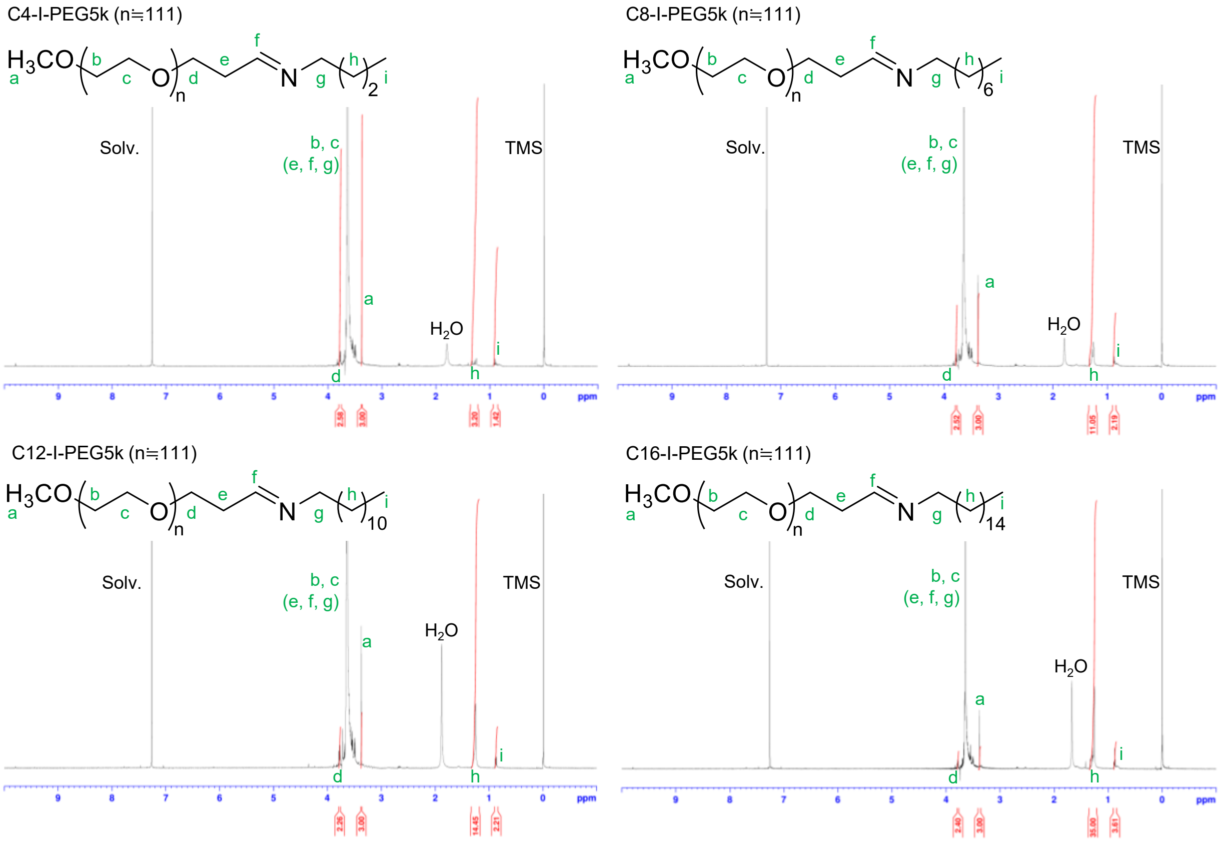

C4-I-PEG5k (n≒111)
C8-I-PEG5k (n≒111)
C12-I-PEG5k (n≒111)
C16-I-PEG5k (n≒111)
Solv.
b, c
(e, f, g)
a
H_2O
TMS
d
h
i
ppm

(D)

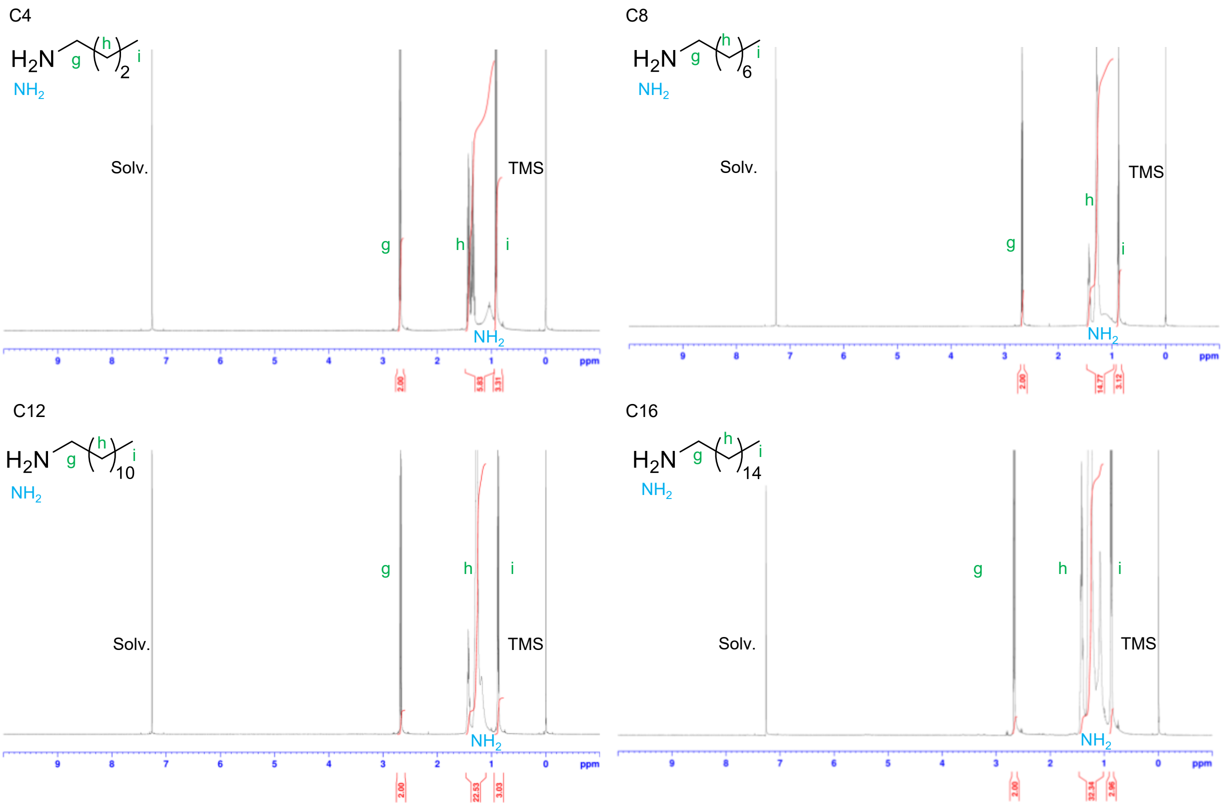

C4
C8
C12
C16
NH_2
Solv.
TMS
g
h
i
ppm

(E)

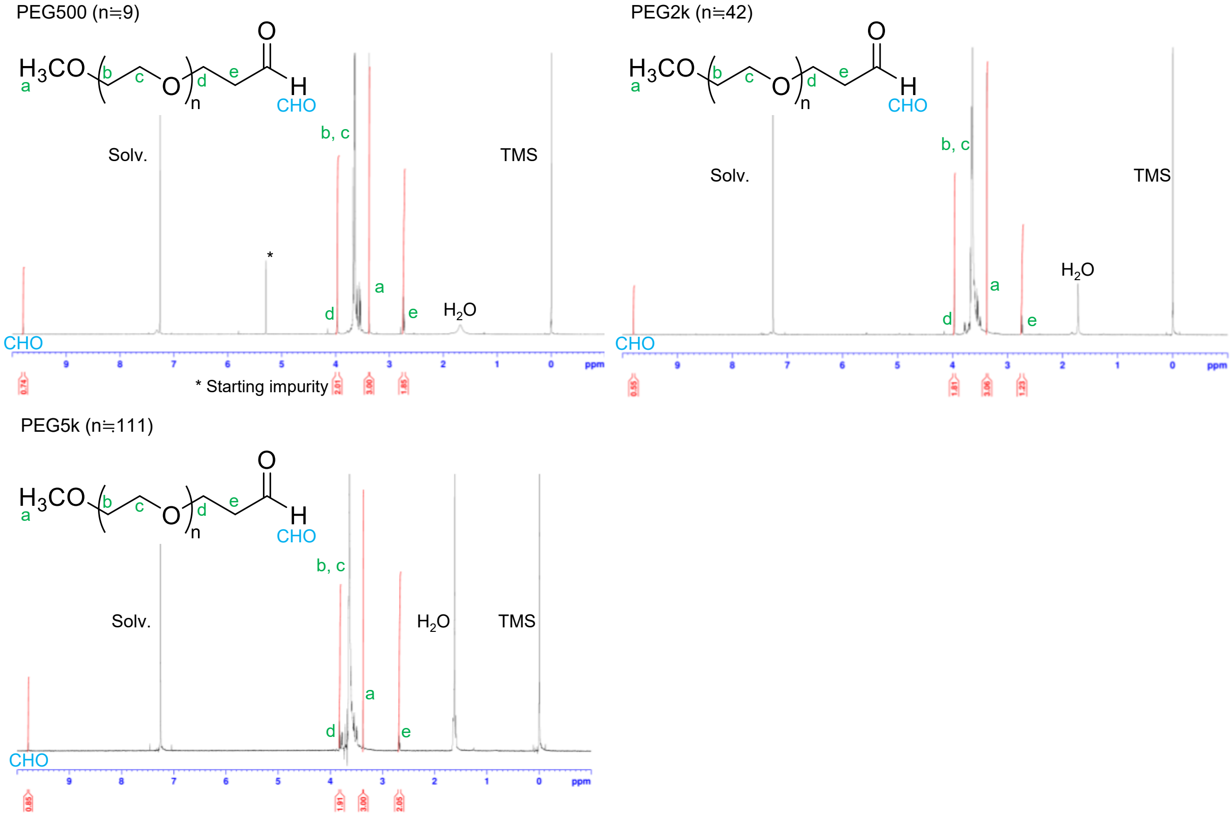

Figure S1. ^{1}H NMR spectra of (A) Cx-I-PEG500, (B) Cx-I-PEG2k, (C) Cx-I-PEG5k, (D) Cx and (E) PEGy (Solv.: $CDCl_3$, 500 MHz).

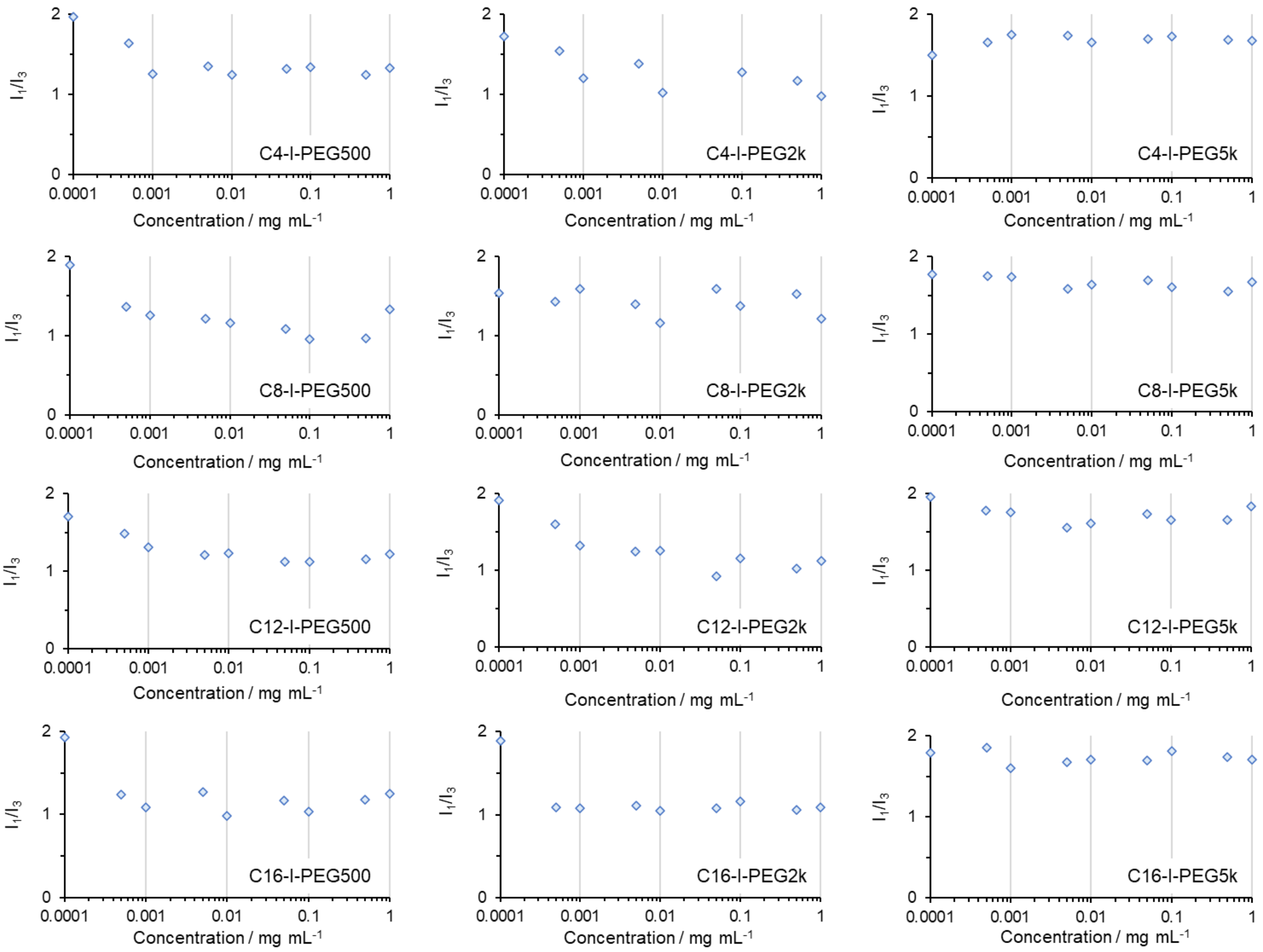

Figure S2. Critical micelle concentration (CMC) measurement of Cx-I-PEGy.

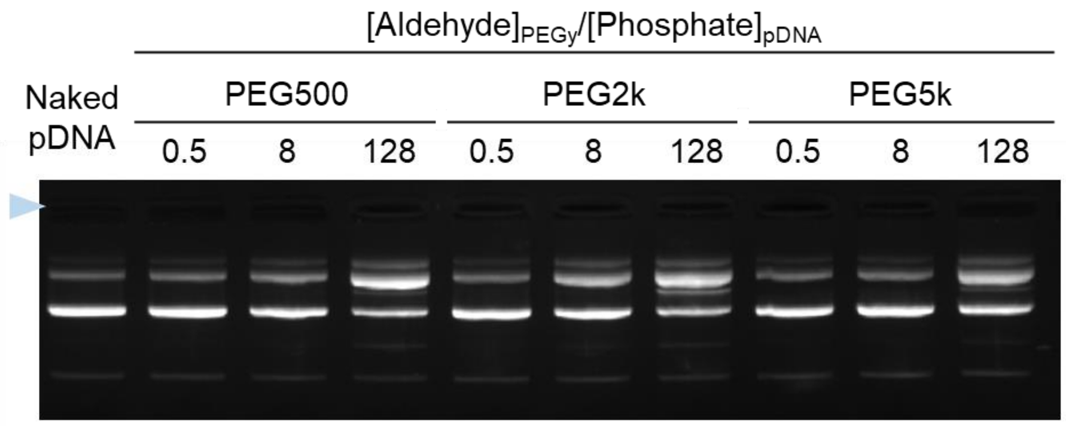

Figure S3. Complex formation between PEGy and pDNA as assessed by agarose gel electrophoresis. The mixing ratios of the aldehyde of PEGy to phosphate group of pDNA, $[\text{Aldehyde}]_{\text{PEGy}}/[\text{Phosphate}]_{\text{pDNA}}$, are indicate. The blue triangle indicates the well where each sample was loaded.

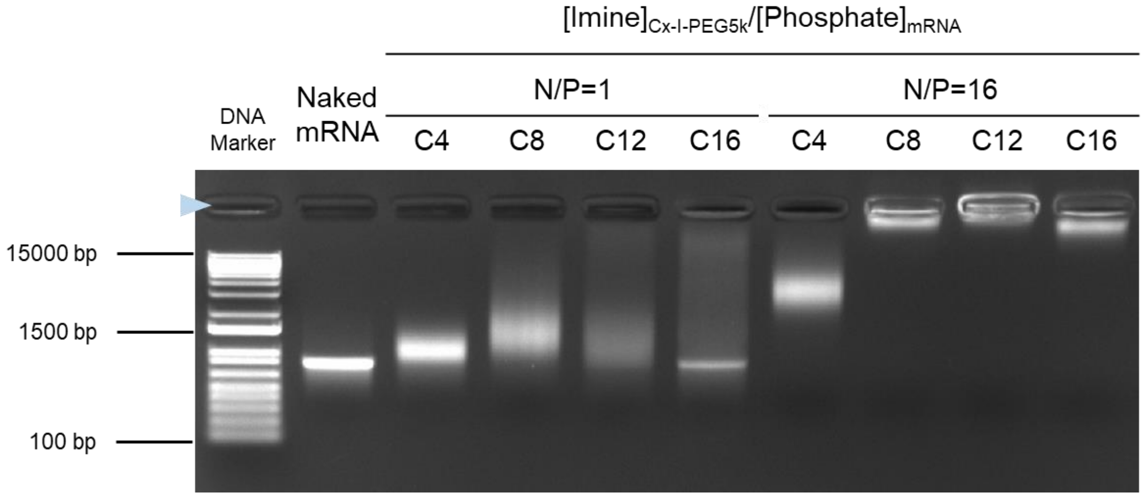

Figure S4. Formation of Cx-I-PEG5k/mRNA complex as assessed by agarose gel electrophoresis. The complexes at a mixing ratios (N/P) of 1 and 16 was incubated for 24 h, followed by loading to the gel. The blue triangle indicates the well where each sample was loaded. For using mRNA, the F-Luc mRNA was purchased from OZ Biosciences (Marseille, France). The mRNA encoded high expression level of firefly luciferase protein. It is modified with 5-methoxyuridine to reduce innate immune responses.

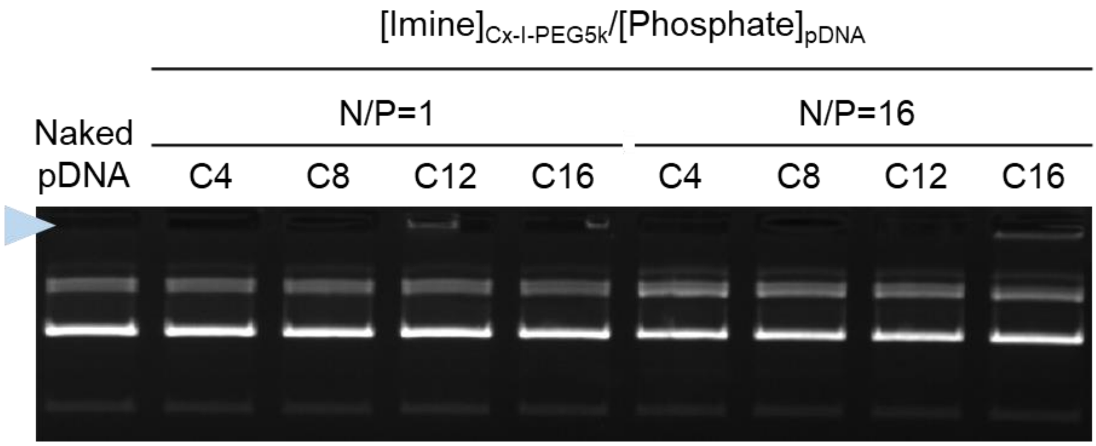

Figure S5. Formation of Cx-I-PEG5k/pDNA I-MICs in phosphate buffer saline (PBS) as assessed by agarose gel electrophoresis. Cx-I-PEG5k and pDNA were incubated at mixing ratios (N/P) 1 and 16 in PBS for 24 h, followed by loading to the gel. The blue triangle indicates the well where each sample was loaded.

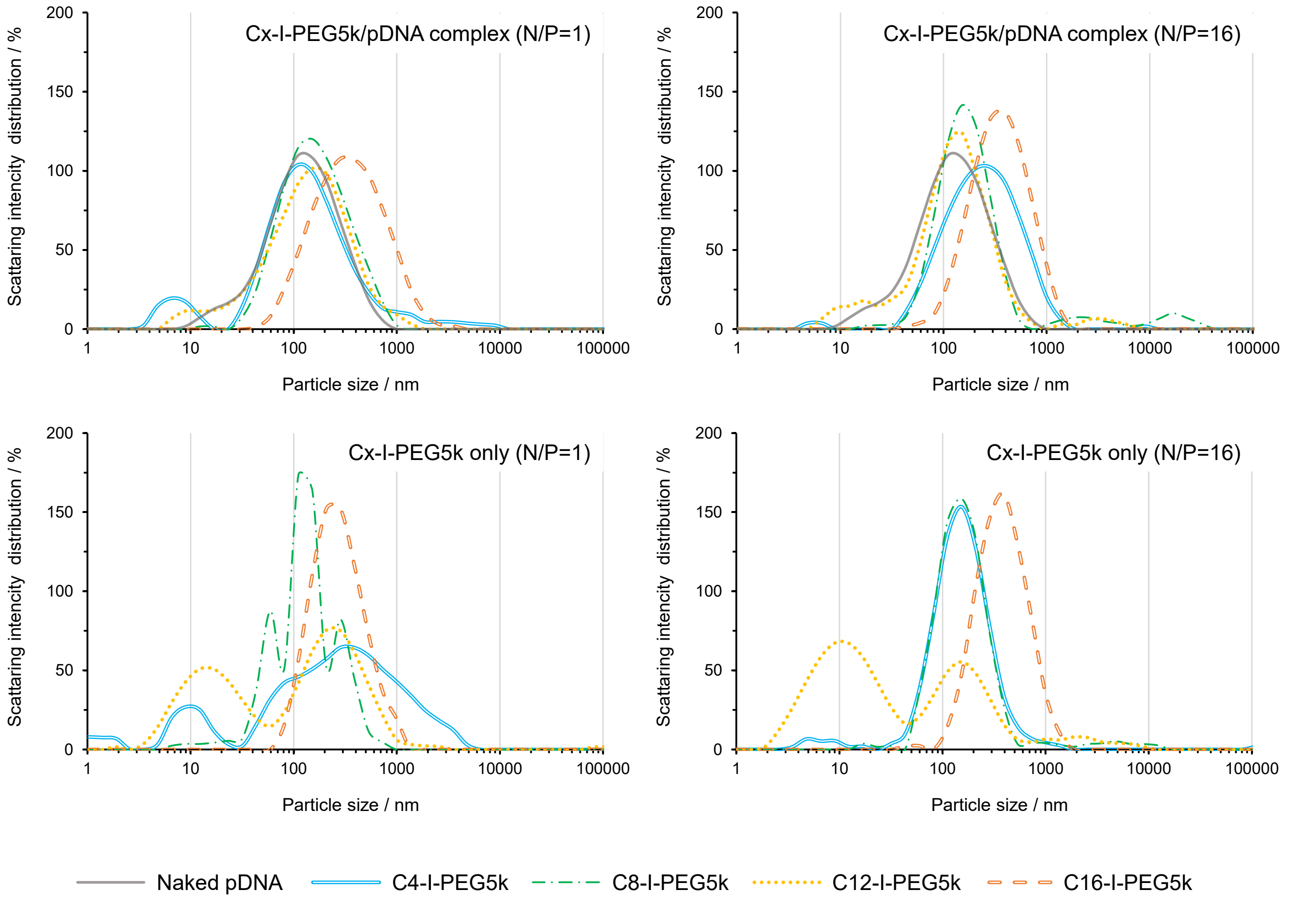

Figure S6. Particle size distribution curves of the Cx-I-PEG5k/pDNA I-MICs. The mixing ratios (N/P) are 1 and 16.

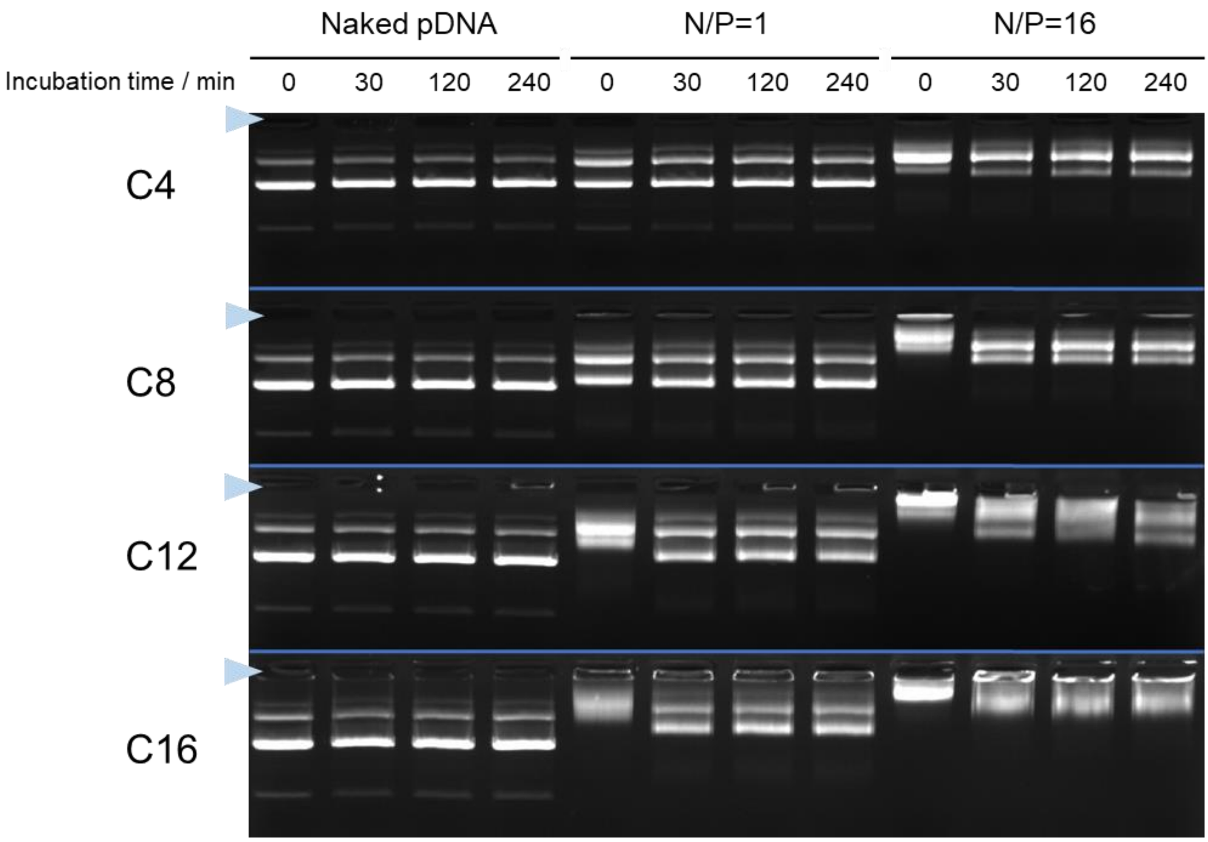

Figure S7. Stability of Cx-I-PEG5k/pDNA I-MICs under physiological ionic strength conditions (in PBS) as assessed by agarose gel electrophoresis. The blue triangle indicates the well where each sample was loaded.

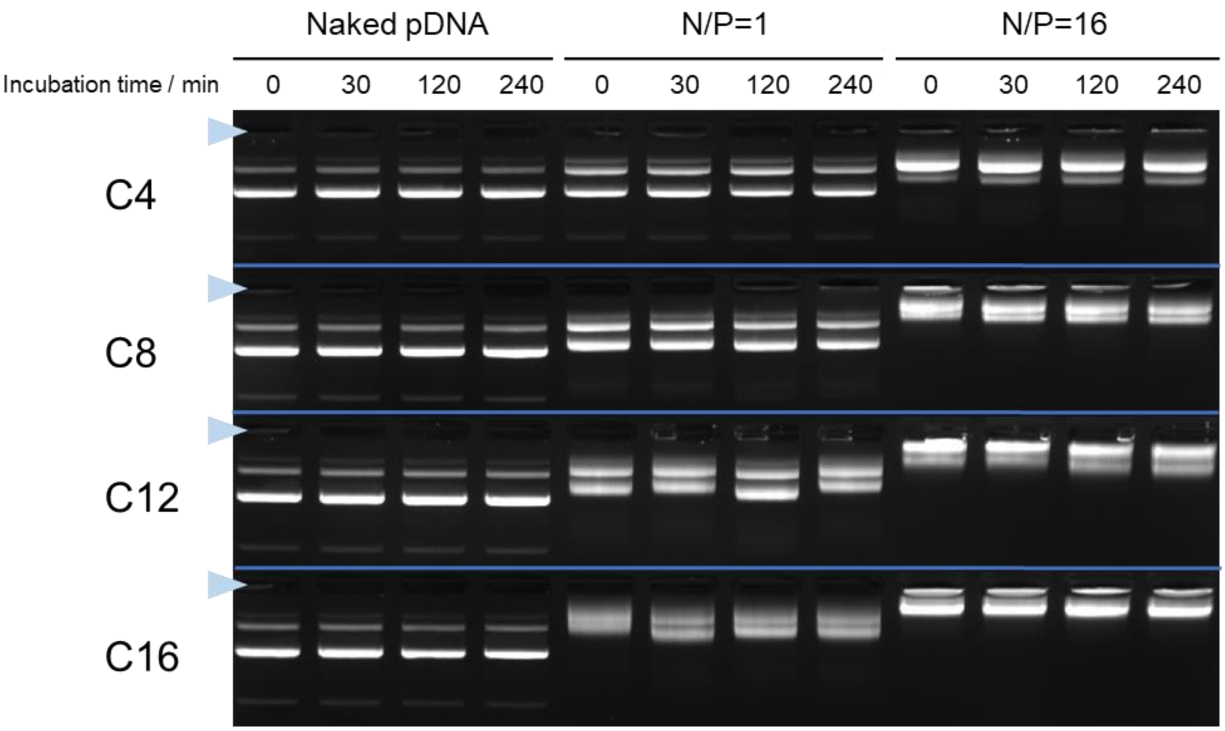

Figure S8. Relase of pDNA from Cx-I-PEG5k/pDNA I-MICs by dextran sulfate (DS) as assessed by agarose gel electrophoresis. The blue triangle indicates the well where each sample was loaded.

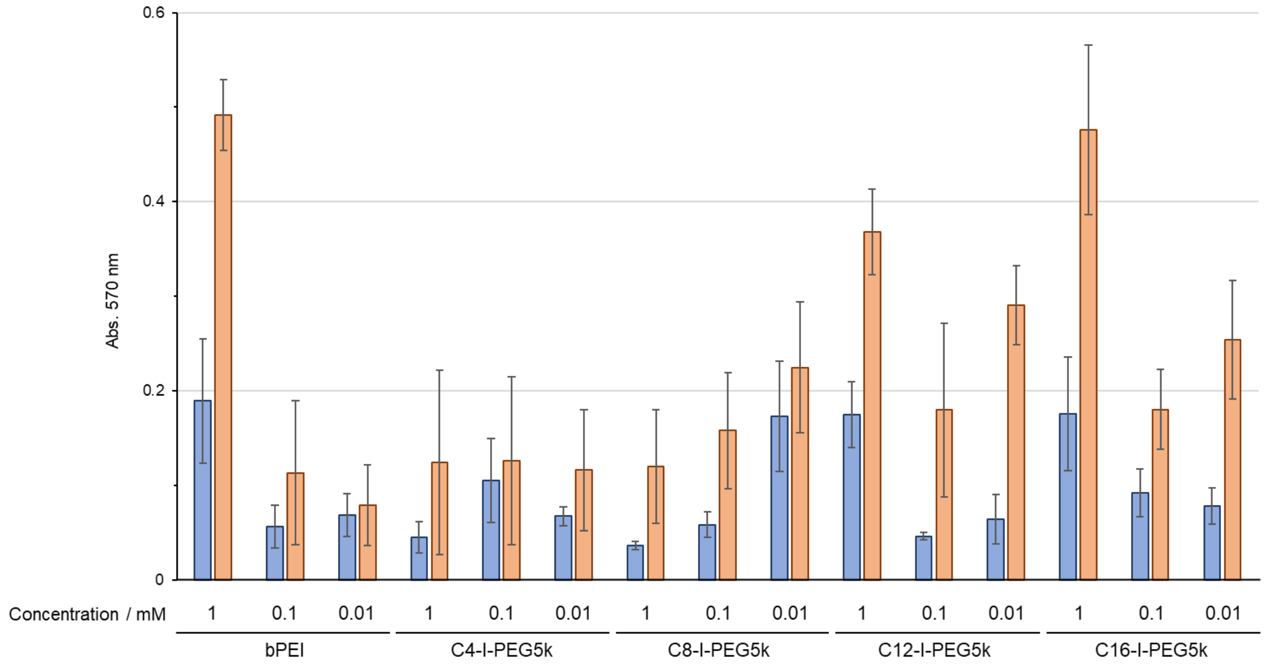

Figure S9. Effect of pH on the hemolytic activity of Cx-I-PEG5k: pH 7 (blue bars) or pH 5 (orange bars).

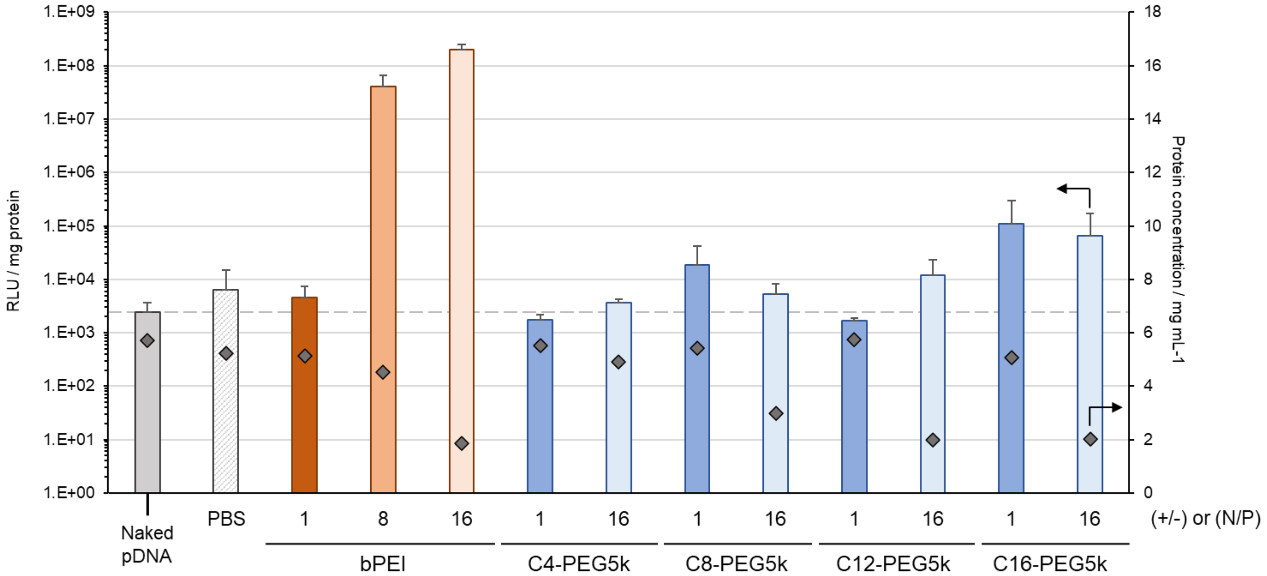

Figure S10. *In vitro* gene transfection efficiency mediated by Cx-I-PEG5k/pDNA I-MICs for C2C12 cells.

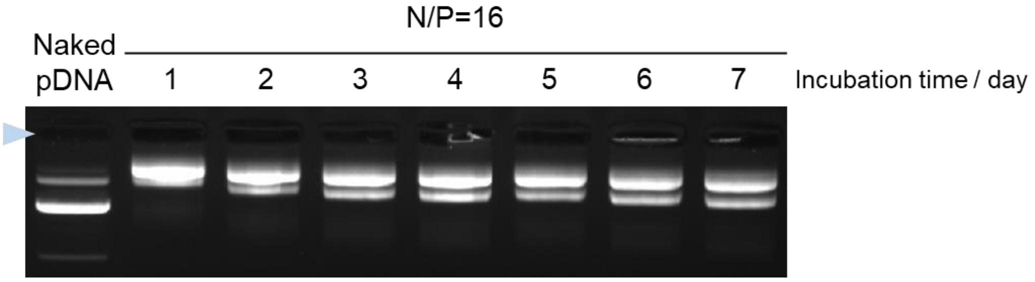

Figure S11. Hydrolysis of C4-I-PEG5k as assessed by agarose gel electrophoresis. pDNA was mixed with each C4-I-PEG5k which was pre-incubated in water for 1-7 days to form I-MICs at mixing ratio (N/P) of 16. The blue triangle indicates the well where each sample was loaded.

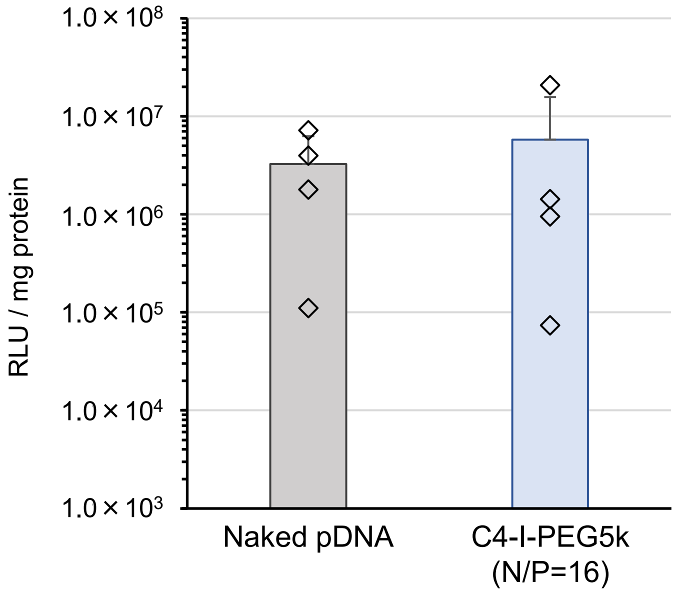

Figure S12. *In vivo* gene transfection efficiency in mouse skeletal muscles. Luciferase gene expression by intramuscular injection of pDNA complex with C4-I-PEG5k after two weeks. The mixing ratios (N/P) is 16. Individual gene expression was determined relative light unit (RLU) normalized by the protein concentration. Rhombic plots were indicated an individual value. All data are represented as the mean and individual values (n=4).

Table S1. Polydispersity index (PDI) of Cx-I-PEG5k/pDNA I-MICs and Cx-PEG5k only. The mixing ratios (N/P) are 1 and 16.

Sample		pDNA	N/P	PDI
Naked pDNA				0.246 ± 0.058
I-MICs	C4-I-PEG5k	+	1	0.251 ± 0.073
		+	16	0.189 ± 0.039
	C8-I-PEG5k	+	1	0.237 ± 0.058
		+	16	0.148 ± 0.011
	C12-I-PEG5k	+	1	0.233 ± 0.040
		+	16	0.200 ± 0.085
	C16-I-PEG5k	+	1	0.280 ± 0.053
		+	16	0.277 ± 0.010
Carrier only	C4-I-PEG5k	-	1	0.380 ± 0.017
		-	16	0.251 ± 0.078
	C8-I-PEG5k	-	1	0.269 ± 0.027
		-	16	0.165 ± 0.054
	C12-I-PEG5k	-	1	0.204 ± 0.033
		-	16	0.122 ± 0.013
	C16-I-PEG5k	-	1	0.256 ± 0.010
		-	16	0.252 ± 0.013
